# Ambulance clinicians’ perspectives on interprofessional collaboration in prehospital emergency care for older patients with complex care needs: a mixed-methods study

**DOI:** 10.1186/s12877-025-05975-w

**Published:** 2025-05-30

**Authors:** Ann-Therese Hedqvist, María Jiménez Herrera

**Affiliations:** 1 Department of Health and Caring Sciences, Linnaeus University, Kalmar, Sweden; 2 Ambulance Service, Region Kalmar, Västervik, Sweden; 3 Nursing Department, Rovira I Virgili University, Tarragona, Catalonia Spain

**Keywords:** Ambulance clinicians, Care plan, Complex care needs, Interprofessional collaboration, Mixed-methods design, Older patients, Prehospital emergency care

## Abstract

**Background:**

Coordinated, multidisciplinary care is essential when addressing the complex needs of an aging population, with prehospital emergency care providers often serving as a common point of contact. Addressing complex care needs while maintaining continuity of care necessitates seamless collaboration between diverse healthcare providers. Despite this, there is limited research on interprofessional collaboration in prehospital care of older patients with complex needs. Understanding what influences interprofessional collaboration and identifying areas for improvement are vital for optimizing prehospital care for this vulnerable population. This study aimed to explore ambulance clinicians' perspectives on interprofessional collaboration in prehospital emergency care for older patients with complex care needs and to identify key factors influencing collaboration.

**Methods:**

An explanatory sequential mixed-methods design was employed in this study, conducted in southern Sweden. In Phase 1, quantitative data were collected via an online survey completed by 118 ambulance clinicians (ACs). Descriptive statistics, chi-square tests, and Kruskal–Wallis tests were used to analyze the data. Qualitative responses were analyzed through inductive content analysis, informing the development of an interview guide. In Phase 2, semi-structured interviews were conducted with 20 ACs and analyzed using inductive content analysis. Findings from both phases were integrated using a joint-display matrix, combining quantitative patterns with qualitative insights for a comprehensive interpretation.

**Results:**

Quantitative findings revealed that although collaboration with patients' families and care staff was generally rated as satisfactory by ACs, significant challenges were reported in coordinating care with other healthcare actors, especially home care nurses. About 89% of respondents reported insufficient access to patient information, highlighting difficulties in retrieving such information. Qualitative data underscored the importance of comprehensive patient information for effective decision-making and alignment with patient preferences and care goals. The integrated analysis identified three key factors influencing interprofessional collaboration: defined goals of care, access to information, and clarity in roles and responsibilities. Challenges in maintaining continuity and responsiveness, particularly during night shifts, were emphasized as barriers to effective collaboration.

**Conclusion:**

Addressing deficiencies in nighttime care coordination, improving access to comprehensive patient information, and strengthening communication pathways between healthcare providers are essential steps in improving interprofessional collaboration to strengthen prehospital care of older patients with complex care needs.

**Supplementary Information:**

The online version contains supplementary material available at 10.1186/s12877-025-05975-w.

## Introduction

The increasing complexity of healthcare needs in aging populations has heightened the importance of coordinated, multidisciplinary care [1–4]. Older patients with multiple chronic conditions or complex care needs often require interventions that span across several levels of healthcare [5–12]. Addressing complex care needs while maintaining continuity of care [13–16] necessitates seamless collaboration between diverse healthcare providers [17, 18]. Prehospital emergency care plays an important role within this continuum of care, serving as a common point of contact during health emergencies [19–23]. In such situations, ambulance clinicians (ACs) are responsible not only for delivering immediate care but also for managing intricate interactions with other healthcare professionals, to ensure continuity and appropriateness of care [24–35].

Ambulance services worldwide face common challenges, including the need to operate in high-pressure, rapidly changing environments. In these settings, collaboration is particularly vital, as patient outcomes often depend on the ability of multiple care providers—from home care nurses to primary care physicians—to work together effectively. Despite the significance of interprofessional collaboration in such contexts, research has largely focused on hospital and primary care settings [36–38], leaving gaps in the understanding of how these dynamics unfold in prehospital environments.

Swedish ambulance services are characterized by a unique model that integrates higher levels of medical expertise within prehospital care teams. Each ambulance team includes at least one registered nurse, typically with specialized training in prehospital emergency care [39]. This expertise not only enables Swedish ACs to make informed decisions regarding patient care pathways but also strengthens collaboration with other healthcare providers. Unlike in many other healthcare systems, where paramedics may have limited autonomy [40–42], Swedish ACs are empowered to assess whether patients can be managed within primary care settings or require hospital transport [39, 43–48]. This approach ensures that decisions about patient care can be aligned with broader care plans, reducing unnecessary hospital admissions and enhancing continuity of care [49–53].

The importance of collaboration for patients, families, and healthcare professionals is well-documented [24, 36–38, 54]. Effective communication and shared understanding between prehospital providers and other healthcare actors are essential for optimizing patient care. However, there remains limited research on how such collaborations function in prehospital settings [55], especially in relation to the management of older patients with complex care needs. Understanding the factors that influence collaboration is vital for improving care delivery, reducing inefficiencies, and ultimately enhancing patient outcomes.

The aim of this study was to explore ambulance clinicians'perspectives on interprofessional collaboration in prehospital emergency care for older patients with complex care needs and to identify key factors influencing collaboration.

The study objectives were:

1. To assess existing collaboration practices of ambulance clinicians and other healthcare actors, identifying any variations based on geographical areas, clinician experience, and education.
2. To explore ambulance clinicians’ access to and perceived need for patient information, examining its impact on the effectiveness of collaboration in prehospital care.
3. To identify key factors influencing collaboration in the prehospital care of older patients with complex care needs from the perspective of ambulance clinicians.

## Clarification of concepts

### Coordinated, multidisciplinary care

Coordinated care refers to the systematic organization and integration of various healthcare services to ensure that patients receive seamless and comprehensive care across different levels and settings [56]. Multidisciplinary care involves the collaboration of professionals from various disciplines—such as physicians, nurses, emergency responders, and social workers—who each bring their expertise to address a patient’s diverse health needs [57, 58]. For older patients with complex care needs, this approach ensures that care is comprehensive, timely, and in line with their overall health goals [2, 59, 60].

### Older patients with complex care needs

This term refers to individuals (65 years or older), who have multiple chronic conditions, such as diabetes, heart disease, and dementia, often accompanied by social or psychological issues [9, 61, 62]. These individuals typically require ongoing care from several healthcare providers and across different settings, including hospitals, primary care, and municipal home care services. Managing such complex cases requires an integrated approach that both medical needs and the broader social and personal factors influencing health and well-being.

### Prehospital emergency care

Prehospital emergency care encompasses all medical care provided before a patient reaches the hospital. In Sweden, this care is primarily delivered by ambulance clinicians (ACs) [39, 63, 64]. While prehospital emergency care often focuses on emergency interventions, it may also include non-emergency patient assessments, referrals to primary care, and decisions on alternative care pathways for patients who do not require immediate hospital treatment. Swedish ACs, particularly registered nurses and specialist ambulance nurses, have the authority to refer patients to community treatment, primary care, or home care services when hospital transport is not deemed necessary.

### Ambulance clinicians (ACs)

In Sweden, ambulance clinicians include specialist ambulance nurses, registered nurses (RNs), and emergency medical technicians (EMTs). Unlike in some other countries, Swedish ambulance services are largely nurse-led, by RNs with additional prehospital training. ACs can independently assess patients, administer medications, and determine the most appropriate level of care [19, 63–67]. Depending on the patient’s condition, ACs may transport them to an emergency department, refer them to primary care, or coordinate follow-up care with home care services. This expanded role means that Swedish ambulance services are increasingly integrated with the broader healthcare system beyond traditional emergency response.

### Home care nurses and care staff

In Sweden, home care nurses (district nurses or municipal nurses) are registered nurses employed by municipal healthcare services. They provide medical care in patients'homes, including medication management, wound care, and palliative care [68, 69]. Home care nurses collaborate closely with primary care physicians and hospital staff to ensure continuity of care.

Care staff refers to assistant personnel who support patients with daily living activities, such as personal hygiene, meal assistance, and mobility. Care staff are typically employed by municipal home care services and do not have formal medical training. Their primary role is to provide social and practical support.

### Interprofessional collaboration

Interprofessional collaboration involves the coordinated efforts of healthcare providers from different professional backgrounds working together to achieve the best outcomes for a patient [54, 70, 71]. In prehospital care, interprofessional collaboration occurs under dynamic conditions requiring timely decision-making, coordination across organizational boundaries, and adaptation to situational demands.

## Methods

### Study design

**Fig. 1:**
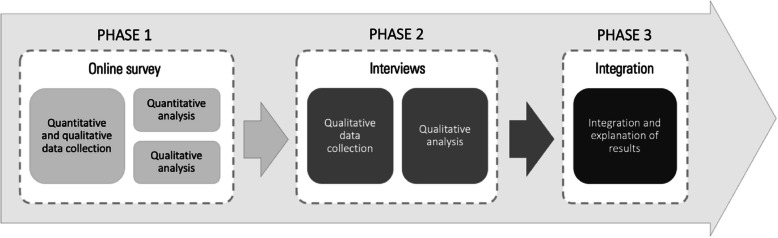
Explanatory sequential mixed-method study design in three phases

An explanatory sequential mixed-methods design [72, 73] was employed. This approach integrates quantitative and qualitative methods to provide a more comprehensive understanding of the research question than a single-method study could achieve [74]. The study design and reporting adhered to the GRAMMS checklist (Appendix 1) for good practice in mixed-methods research [75]. The study unfolded in three phases (Fig. 1):

- Phase 1: An online survey was conducted to gather broad insights from respondents, incorporating both quantitative and qualitative questions [76].
- Phase 2: A protocol for semi-structured interviews was developed based on the survey results and interviews were conducted to gather rich contextual data [73].
- Phase 3: The data from Phases 1 and 2 were integrated to form a holistic understanding of the findings [77].

### Study setting

The study was conducted in a region of southern Sweden with a population of approximately 250,000 residents. This region includes a variety of geographical settings such as urban centers, rural landscapes, an extensive archipelago, and sparsely populated areas. Notably, the area has a significant proportion of older adults, making it an ideal setting for investigating prehospital emergency care and interprofessional collaboration for older patients with complex care needs.

Elderly care in Sweden is structured to provide comprehensive and coordinated services that enable older individuals to live independently in their homes for as long as possible [68]. The multidisciplinary team around the patient includes home care nurses, who manage medical care and monitor chronic conditions; care staff, who support daily living activities; primary care physicians, responsible for overall medical management and medical care plans; primary care nurses, providing advanced care in areas such as palliative and chronic disease management. Family members also play a crucial role by providing support and assisting with daily activities.

The ambulance care organization in Sweden is regulated by the Health and Social Services Act [78], and each of the 21 regions is responsible for providing ambulance care to its residents. The selected region manages about 38,000 ambulance assignments annually, supported by 15 ambulance stations. The Swedish ambulance service is well-developed and characterized by a high level of competence and specialized training. ACs in Sweden come from diverse professional backgrounds and have varying levels of education and training. The primary roles within the ambulance service are those of RNs and EMTs. Many RNs have specialized training in prehospital emergency care, often holding a bachelor’s degree in nursing and additional certifications or postgraduate training in emergency and prehospital care. These specialized RNs are equipped to handle complex medical emergencies and provide advanced life support. EMTs, on the other hand, typically complete a vocational education program focused on emergency medical services. They are trained to provide basic life support, assist in patient stabilization, and perform essential prehospital procedures.

### Recruitment and participants

Participants were recruited through purposive sampling, where all ACs employed in the region’s ambulance service were invited to participate voluntarily. The participants in this study were ACs, including both RNs and EMTs, working in the region’s ambulance service. The Survey System calculator (https://www.surveysystem.com/sscalc.htm) was used to determine the sample size needed to provide a margin of error of 6% with a 95% confidence interval. This was found to be 115 ACs. A total of 200 ACs were invited, and 118 responded, yielding a response rate of 59%. For the qualitative phase, information power was estimated to be achieved with 15–20 interviews [79].

With authorization from the regional ambulance service manager, an online survey detailing the study was distributed to all ACs within the region. Submission of a completed survey was regarded as providing consent to participate in the study. For the subsequent interview phase, participants were strategically selected from the survey respondent pool to ensure representation across various station locations and a range of experience levels. Informed consent was obtained from each participant prior to conducting their interview.

### Data collection

Data were collected in two distinct phases from November 2020 to April 2022.

### Phase I

The online survey (Appendix 2) was initially pilot tested with two individuals, refined based on their feedback, and then emailed to all ACs in the region. The survey aimed to assess current collaboration practices and access to information, utilizing a 4-point Likert scale without a neutral midpoint to elicit clear positions [80, 81]. This approach was chosen to encourage respondents to indicate either adequacy or insufficiency in collaboration rather than selecting a neutral stance. The scale ranged from'inadequate'to'complete,'with responses of 1–2 considered as indicating insufficiency and 3–4 as indicating adequacy. Additionally, open-ended questions were included to capture narrative data on collaboration experiences.

### Phase 2

Semi-structured interviews [82] were conducted, guided by an interview guide (Appendix 3) developed from the survey results to explain the replies in the survey and explore key factors influencing interprofessional collaboration. The initial survey results informed the interview topics [83], which were further refined through two pilot interviews. This ensured the relevance of the questions and the effectiveness of the interview technique. One pilot interview was of sufficient quality to be included in the final dataset. Interviewees were given the option of face-to-face or telephone interviews, each lasting between 25 and 70 min. All interviews were recorded and transcribed verbatim for analysis.

### Analysis

#### Phase 1 – Survey data analysis

Quantitative survey data were analyzed using descriptive statistics to identify trends and distributions. Statistical analyses and visualizations were performed using R software (version 4.1.0) and IBM SPSS Statistics version 26. Relationships between variables, such as geographical area, education, and experience (categorized into five groups in Table 1), were investigated using chi-square tests and cross-tabulations. Analyses of Likert scale and categorical data aimed to correlate demographic and professional backgrounds with experiences of collaboration. The Kruskal–Wallis test was employed for variables with more than two groups, with statistical significance set at *p* < 0.05.

Qualitative survey responses were analyzed through inductive content analysis [84]. Initial coding led to the abstraction of categories, which informed the development of the interview guide for Phase 2.

#### Phase 2 – Interview data analysis

Interview transcripts underwent inductive content analysis [84]. The process involved data immersion through repeated readings and memo creation, followed by systematic open coding, where meaning units were identified and labeled. These codes were then grouped into subcategories and main categories, ultimately leading to the formation of a primary category. This process followed the approach outlined by Elo and Kyngäs [84] for inductive content analysis. Brief memos were composed throughout the analysis to aid the analytical process.

#### Phase 3 – Data integration

In accordance with the explanatory sequential mixed-methods design [72, 73], the integration phase adopted a “building” approach to data collection and analysis, where the interview questions were directly shaped by the survey findings [83]. Quantitative data outlined broad patterns, whereas qualitative data provided a nuanced understanding of detailed experiences. Data from both phases were analyzed and synthesized. The integrated results were presented in a joint-display matrix, providing a comprehensive interpretation of the combined dataset.

### Ethical considerations

The study has been coherent with the ethical guidelines in the Declaration of Helsinki and all participation was individual and voluntary with informed consent acquired. No personal or sensitive data were collected. Under Swedish regulations, ethical approval was not necessary for the survey, as it was part of a quality improvement project. However, ethical approval was obtained from the Swedish Ethical Review Authority (Registration number 2020–01219) for the interviews with ACs.

## Results

The results are presented in four subsections: study population, quantitative findings from the survey, qualitative findings from free-text answers in the survey and interviews, and integration of the combined insights.

### Study population

Survey responses were obtained from 118 ACs, providing a representative sample of the demographic distribution within the regional ambulance service. The education levels were RNs specialized in ambulance care, RNs with other specializations, RNs without any specialization, and EMTs. The average experience within ambulance service among survey respondents was 17 ± 11 years, with a median of 15 years. For a detailed demographic breakdown, refer to Table 1.

**Table 1:** Demographics of the 118 survey respondents

Variable	n (%)
Education level	
RN with specialization in ambulance care	76 (64.4)
RN with other specialization	8 (6.8)
RN without specialization	19 (16.1)
EMT	15 (12.7)
Number of years’ experience in ambulance service	
0–5	23 (19.5)
6–10	17 (14.4)
11–15	21 (17.8)
16–20	21 (18.8)
> 20	36 (30.5)
Geographical area	
North	31 (26.3)
Middle	22 (18.6)
South	42 (35.6)
Island	13 (11.0)
Not specified	10 (8.5)

*RN* Registered nurse, *EMT* Emergency medical technician

### Quantitative findings

#### Assessment of current collaboration practices

This study aimed to assess the existing collaboration practices between ACs and various healthcare actors, evaluating how these relationships were perceived across different settings and geographical areas. The overall mean assessment of collaboration practices between ACs and various healthcare actors indicated that approximately 73% of interactions were perceived as satisfactory (ratings of 3 or higher).

A majority of ACs rated their collaboration practices as satisfactory when interacting with patients'spouses (97%) and children (89%). Positive interactions were also noted with care staff, both during the day (86%) and at night (80%). Collaboration with primary care nurses was rated as satisfactory by 76% of respondents, whereas 62% expressed satisfaction with their collaboration with home care nurses during the day. However, only 51% of respondents found their collaboration with primary care physicians to be satisfactory, and collaboration with home care nurses at night received the lowest satisfaction rating, at 41%. It is important to note that some respondents reported having no experience of collaboration with certain healthcare actors: primary care physicians (*n* = 7), primary care nurses (*n* = 3), home care nurses during the day (*n* = 1), and home care nurses at night (*n* = 1). These respondents were excluded before calculating the percentages above.

The analysis revealed that the level of education did not significantly affect collaboration assessments among ACs. In contrast, the number of years of experience in the ambulance service had a notable impact on the rated collaboration with care staff at night and home care nurses at night. Specifically, ACs with over 20 years of experience gave the highest ratings for collaboration with care staff at night (*p* = 0.012) and home care nurses at night (*p* = 0.042). ACs with 6–10 years of experience gave the lowest ratings for such collaborations.

The survey also assessed collaboration across different geographical areas, revealing significant variations (refer to Table 2). Disparities were particularly evident regarding collaboration with care staff, home care nurses, and primary care nurses, with ACs from island regions giving higher ratings than those from central and northern areas. For care staff during the day, the island region had the highest mean rank (83.88), indicating a higher rating of collaboration compared with the middle region (mean rank = 49.14). Similarly, for care staff at night, the island region reported a higher mean rank (89.42) than the north region (47.98), suggesting better collaboration at night in the island region (*p* = 0.001). Significant regional variations were also observed in the quality of collaboration with home care nurses during both day and night. The island region consistently reported the highest ratings, with mean ranks of 81.54 during the day and 83.19 at night, whereas the north region reported the lowest ratings, with mean ranks of 43.55 and 46.60, respectively. Additionally, the significant difference in mean ranks (*p* = 0.002) indicates that collaboration with primary care nurses was better in the island region (mean rank = 83.88) than in the north region (mean rank = 43.95). These findings highlight the influence of geographical location on interprofessional collaboration practices, with the island region consistently showing more favorable ratings than other regions, particularly the north.

**Table 2:** Assessment of collaboration practices with different actors, by geographical area

Rated collaboration with different actors, mean rank								
Geographical area	Care staff, day	Care staff, night	Home care nurse, day	Home care nurse, night	Primary care nurse	Primary care physician	Spouse	Child
North	53.63	47.98	43.55	46.60	43.95	48.45	58.30	57.90
Middle	49.14	54.09	64.52	51.75	55.57	49.91	52.64	55.69
South	61.83	59.10	61.04	61.57	61.14	59.92	59.32	58.38
Island	83.88	89.42	81.54	83.19	83.88	75.19	48.15	45.46
Not specified	59.00	69.90	56.67	71.40	60.45	50.70	53.50	45.46
Kruskal–Wallis H	12.913	19.661	14.960	15.394	17.097	8.843	2.096	2.554
p	.012*	.001*	.005*	.004*	.002*	.065	.718	.635

*Statistically significant at *p* <.05

#### Information needs and access to patient care plans

**Fig. 2:**
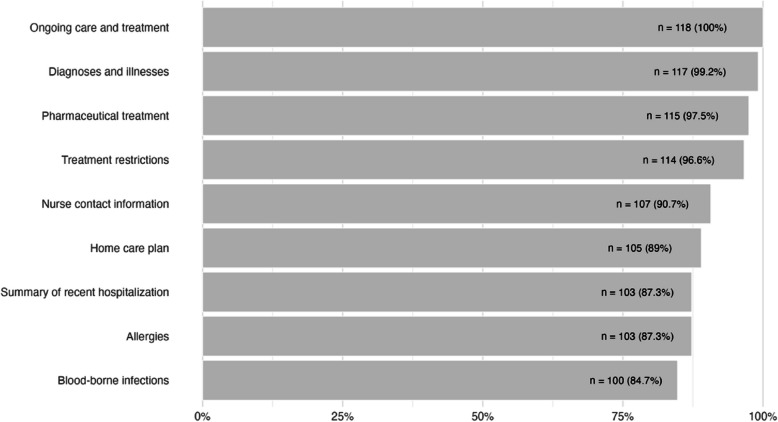
Distribution and frequencies of patient information requested by ambulance clinicians (*n* = 118)

Figure 2 presents the distribution and frequencies of various types of patient information requested by the survey respondents. The most frequently requested information related to ongoing care and treatment, with every respondent (100%) indicating its importance. This was closely followed by diagnoses and illnesses (99%) and pharmaceutical treatment details (98%). Treatment restrictions were identified as necessary by 97% of respondents, whereas nurse contact information was sought by 91%. Additionally, 88% of respondents expressed the need for access to the patient’s home care plan. Information on recent hospitalization and allergies were both requested by 87% of respondents. The least requested information related to blood-borne infections, with 85% indicating this need. Each type of requested information was statistically significant according to the chi-square goodness of fit test, with χ2(1, *N* = 118) values ranging from 114.03 to 56.98, all within a 95% confidence interval (*p* < 0.05).

Access to a patient's care plan was considered relevant by 98% of respondents, with significant variations depending on educational level (*p* = 0.0017). This result was determined using Fisher's exact test. Among the respondents, RNs without specialization and RNs with specialization in ambulance care found it most relevant to have access to the care plan, with 100% indicating either"yes"or"maybe."More specifically, RNs without specialization had 79% indicating"yes"and 21% indicating"maybe,"whereas RNs with specialization in ambulance care had 75% indicating"yes"and 25% indicating"maybe."For EMTs, 93% indicated either"yes"(57%) or"maybe"(43%), whereas 7% indicated"no."For RNs with other specialization, 75% indicated either"yes"(67%) or"maybe"(33%), whereas 25% indicated"no."Experience in years did not significantly affect this preference.

**Fig. 3:**
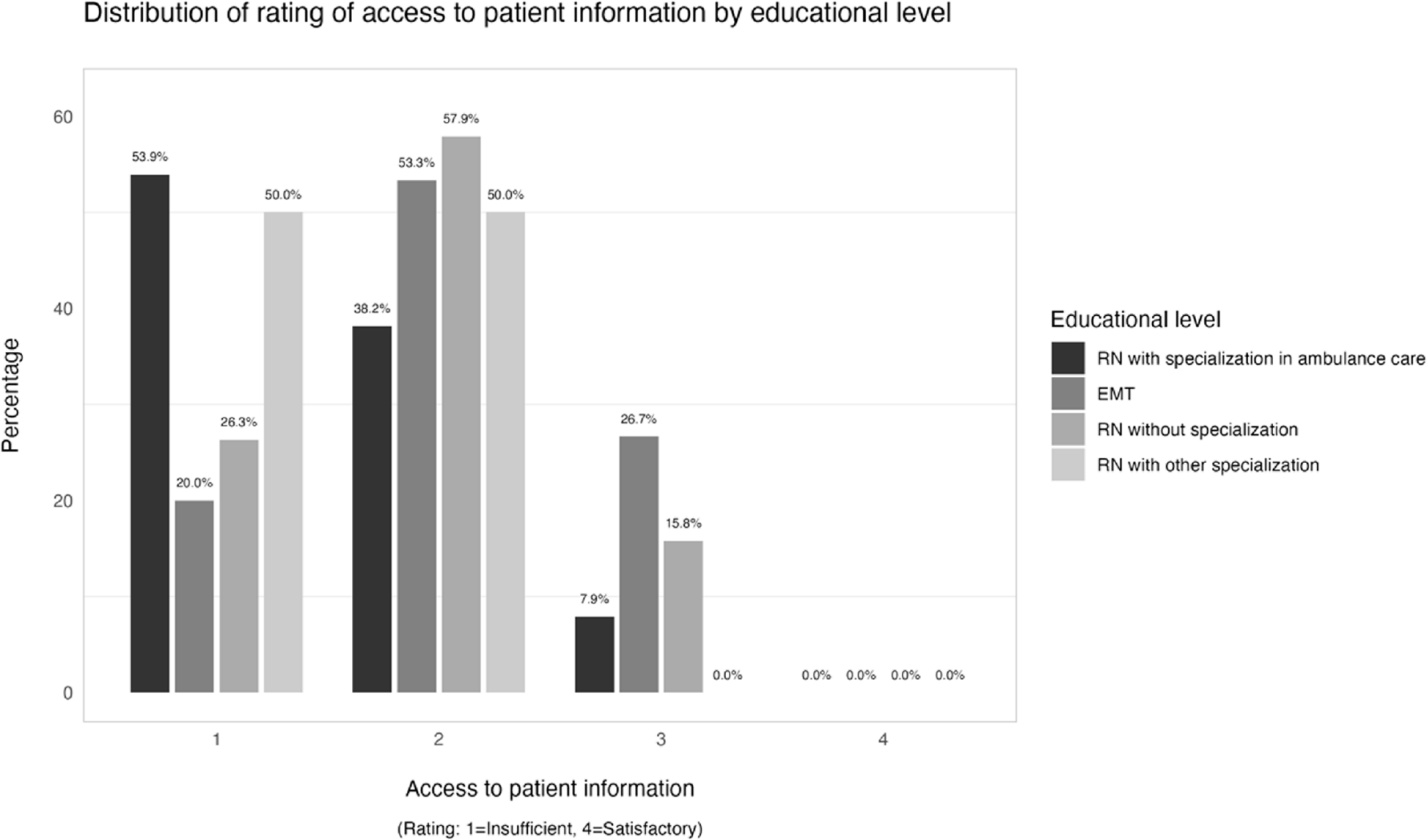
Distribution of ratings of access to patient information by educational level

Eleven percent of respondents rated their current access to patient information as satisfactory, with a score of 3 or higher. The distribution of ratings of access to patient information by educational level is visualized in Fig. 3. When evaluated using Fisher's exact test, no statistically significant correlation was found between access to patient information and educational level, years of experience, or geographical location.

### Qualitative findings

The analysis of open-ended survey responses identified several key factors influencing interprofessional collaboration, which were categorized into external and internal domains, as detailed in Table 3. External factors included challenges related to the documentation of care plans, availability of resources, staff continuity and competence, and timing constraints. Internal factors highlighted issues such as difficulties in accessing patient information, deficiencies in knowledge and decision support, and a lack of clarity regarding roles and responsibilities.

**Table 3:** External and internal factors impacting interprofessional collaboration as revealed by open-ended survey responses

Domain	Category	Description	Examples of responses
**External factors**	Documented care plans	Availability of comprehensive patient care plans and medical histories is especially important when patients are unable to advocate for themselves, as the lack of such information can lead to default emergency transports that may not be the most appropriate course of action	“Information about the care plan, current and underlying conditions. That no one really knows what's decided or is going on.” “Often unclear plan around the patient. There is no documented plan for the current situation. These patients often find it difficult to speak for themselves, which is why it is so important that the plan is documented somewhere.” “Making the best decision about whether to transport a patient to a care facility or to allow them to remain at a place like a nursing home hinges critically on the information we have at hand upon arrival.” “As a'safety measure'due to insufficient information, the multimorbid elderly person will often be transported to the emergency department."
	Resources and availability	The limited availability of on-site home care nurses is considered to contribute to reliance on ambulance services as a substitute. The lack of home care nurses and physicians for the elderly was identified as a key factor influencing decision-making and the potential overuse of hospital transports	“Home care nurses are often not present on site.” “I feel that the home care service does not get the help they need from home care nurses and therefore contacts the ambulance service instead.” “You often get the answer'the nurse didn't have time to come here.'More home care nurses would be good, as well as physicians dedicated to the elderly, who are not fully booked with other patients, so perhaps more people could be referred to health centers for assessment.” “Availability is important for us to be able to have a good collaboration and have the patient in focus.”
	Competence and continuity	Resource availability and staff competence emerged as pivotal, with inconsistencies in staff familiarity affecting patient care continuity. Permanent staff's familiarity with patients enhanced care, whereas temporary staff's limited knowledge often led to over-triage, particularly when assistant nurses faced assessments beyond their expertise, despite their best efforts	“On [name of island], the staff is more permanent and knows the patients well, which facilitates assessment, treatment, and coordination—that the patient gets to the right level of care. However, in other areas, there is more temporary care staff and then it is more difficult to get crucial information that affects my assessment of the patient, which can lead to the patient being transported to a higher level of care than they may need.” “Care staff frequently find themselves isolated in making assessments and may lack the necessary knowledge, despite their best efforts.”
	Timing	The quality of care coordination is significantly influenced by the time of day, with better management during daytime due to higher staff availability and greater patient familiarity, and decreased effectiveness at night due to informational and commitment gaps	“During the day, they are very helpful, and it is noticeable that the old people have great confidence in the care staff in the home care service. At night, it is worse, as they do not know the patients and have less commitment—then our work becomes more difficult.” “It is much easier to coordinate care during office hours. The problems come when things happen at night, and we don’t have any background information.”
**Internal factors**	Access to patient information	Immediate access to patient medical records is essential for ambulance clinicians to conduct informed assessments, a feature implemented in certain areas but missing in others	“We on [name of island] have a well-developed local healthcare system, but in other areas there is usually a lack of background information and planning documentation for the elderly. It would be easier if we had access to the patient's medical records on site at the patient’s home.” "As we do not have access to medical records when we are with the patient, we cannot get an overall picture of the patient and then we find it difficult to make an accurate assessment.”
	Knowledge and decision support	There is a perceived gap in geriatric expertise among ambulance clinicians, with a need for better decision support systems tailored to the complexity of multimorbidity in the elderly, beyond comparisons to younger, healthier patient profiles	“The question is whether we have full geriatric knowledge to fully apply good quality care that leads to increased patient safety.” “Knowledge is lacking in the multifaceted problem of multimorbidity, countless medicines, with all the possible interactions. The older person's atypical symptom image. A lack of decision support. It is not sustainable for the elderly, with a need for assessments other than against a healthy young man.”
	Definition of roles and responsibilities	Ambulance clinicians question the clarity of their roles—whether they are emergency responders for critical cases or assessors, directing patients to the correct level of care. This ambiguity affects both resource allocation and the efficacy in meeting patient needs	“If there is time, I usually think that we have the conditions to find the right level of care for the patient.” “Are we emergency professionals for priority 1 assignments or are we assessment units for finding the right level of care? If the assignment is to assess and find the right level of care, a lot of resources are taken away from priority 1 assignments.”

Building on the survey findings, interviews were conducted with 20 ACs, to gain deeper insights into these issues. The survey responses indicated that EMTs were not primarily responsible for making crucial medical decisions in the prehospital setting. Therefore, the choice was made to focus the interviews on RNs, who play a more central role in clinical decision-making. This approach ensured that the interviews captured the perspectives of those most directly involved in the complexities of patient care. The average experience in ambulance service among interview participants was 12 ± 8 years, with a median of 13 years. The demographics of the interview participants are outlined in Table 4.

**Table 4:** Demographics of the 20 interview participants

Variable	n (%)
Gender	
Woman	12 (60)
Man	8 (40)
Age (years)	
29–35	7 (35)
36–42	3 (15)
43–49	5 (25)
50–	5 (25)
Education level	
RN with specialization in ambulance care	14 (70)
RN with other specialization	1 (5)
RN without specialization	5 (25)
Number of years in ambulance service	
0–5	5 (25)
6–10	5 (25)
11–15	5 (25)
16–20	2 (10)
> 20	3 (15)
Geographical area	
North	16 (80)
Middle	0 (0)
South	3 (15)
Island	1 (5)

*RN* Registered nurse

The analysis of the interviews resulted in three categories: "Preconditions and dependencies," "Building collaborative relationships," and "Co-creating patient care." These categories, along with subcategories and descriptions, are presented in Table 5.

**Table 5:** Categories, subcategories, and descriptions from the findings of phase 2

Category	Subcategory	Description
Preconditions and dependencies	Availability	Effective collaboration requires the availability of professionals across disciplines, extending beyond physical presence to engagement in consultations and decision-making processes
	Continuity	Continuity ensures that patient care does not get disrupted and there is a seamless flow of services with professionals consistently involved to provide uninterrupted care, even during staff changes
	Information	Access to relevant patient data for all team members is essential, necessitating a system that facilitates timely and secure data sharing
	Knowledge	Each professional brings a unique expertise to the table. Recognizing and respecting each other's knowledge is critical. Opportunities for knowledge dissemination through interprofessional learning can enhance collaboration
Building collaborative relationships	Relation	Building a foundation of trust is vital in a collaborative relationship, with a clear understanding of each member's roles, strengths, and limitations
	Co-operation	Effective collaboration goes beyond understanding roles to an active willingness to cooperate, incorporating compromise, receptiveness to feedback, and joint efforts towards common goals
	Recognized pathways	The establishment of clear, recognized processes and protocols streamlines operations and delineates responsibilities and actions for different scenarios
	Communication	Open, transparent, and efficient communication is important to prevent misunderstandings and foster effective collaboration
Co-creating patient care	Mutual understanding	A collective understanding of shared goals, patient needs, and the overarching vision of care is fundamental to designing and implementing effective care plans
	Internal resources	Utilizing the team members'tools, skills, and knowledge to complement each other, fill gaps, and enhance care quality
	External resources	Recognizing when to engage external resources and integrating them effectively into patient care is crucial for optimal outcomes

#### Preconditions and dependencies

ACs reported significant constraints due to the variable availability and expertise of other collaborators. Key dependencies included the on-site presence of home care nurses and the consistency of knowledgeable care staff. A notable concern was the limited patient familiarity among care staff at night:

*“Care staff during night shifts are less informed, as they usually see patients when they're least active. As a result, they often lack detailed information to pass on, which means you're left without any solid info. It's hard to get the full picture, and nobody's there to fill you in. That's a big hurdle, if you ask me.” (P14)*

The importance of direct communication with home care nurses was acknowledged, yet the rarity of such interactions led ACs to frequently compensate for this gap, emphasizing the need for shared medical records:

*"It would be helpful if we could see what the other care providers had written, and what is decided for this patient. What is said about the patient’s care plan?” (P18)*

Furthermore, ACs emphasized the necessity of detailed information on a patient's medical history and current treatments, especially when symptoms were ambiguous, to guide the decision-making process. Despite these challenges, clinicians relied on standard protocols in emergencies involving critically ill older patients, focusing on present symptoms to deliver immediate care.

#### Building collaborative relationships

Establishing interprofessional collaboration is rooted in cultivating strong, trust-based relationships and developing clear, mutually recognized protocols that transcend professional boundaries. This aspect of collaboration is particularly important in smaller communities, where personal familiarity with other healthcare providers can significantly enhance the collaborative dynamic. In such environments, the interpersonal connections between ACs and other care providers are cornerstones of effective teamwork:

*“In our smaller community, there's better rapport between the care providers. Recognizing each other's faces and understanding individual roles helps streamline interactions and build a stronger collaboration.” (P15)*

Such relationships foster a sense of shared purpose and make it easier to establish reliable communication channels—an integral aspect of coordinating care. Here, relationships and cooperation are intertwined, as personal connections foster a willingness to engage and compromise, enhancing teamwork. Recognized pathways or standardized procedures then offer a structured approach, establishing clear procedures and roles that everyone understands and respects. This clarity is essential for ensuring that all team members are aligned in their objectives and methods. Communication acts as the glue that holds the collaborative effort together. Open, transparent dialogue enhances the mutual understanding necessary for collective decision-making and the efficient coordination of patient care.

#### Co-creating patient care

The concept of co-creating care reflects a unified approach to healthcare delivery, emphasizing mutual understanding, shared goals, and collective responsibility in managing the care of older patients with complex needs. A sentiment shared among ACs highlights the importance of a united front:

*"To effectively care for our patients, we must work together with a shared vision and understanding. It's about pooling our strengths and resources for the greater good.” (P16)*

Mutual understanding underscores the importance of all care providers, the patient, and their family, being aligned in their care objectives and strategies. Such alignment ensures that care decisions are made with a comprehensive view of the patient's needs and preferences. Internal resources refer to the capabilities and expertise within the healthcare team that can be leveraged to provide patient-centered care. Utilizing these resources effectively requires a collaborative mindset and the ability to integrate diverse skill sets and knowledge bases. Use of external resources involves recognizing when additional support is needed beyond that available in the immediate healthcare team and knowing how to incorporate such resources into the patient's care plan seamlessly. These resources may include specialists or technical solutions that enhance care delivery.

### Integrated results

#### Key factors influencing interprofessional collaboration

The integrated analysis revealed three key themes that influence interprofessional collaboration in prehospital emergency care for older patients with complex needs (refer to Table 6). These were: “Defined goals of care,” emphasizing the importance of shared understanding of patient care objectives; “access to information,” highlighting the necessity of timely and accurate patient data for informed decision-making; and “clarity in roles and responsibilities,” underscoring the need for well-defined roles to ensure effective and coordinated care.

**Table 6:** Integrated findings matrix with quantitative and qualitative results and the integrated insights

Quantitative results			Qualitative results		Integrated results
Aspect	n (%)	Summary	Exemplar quotation	Summary	Theme and insights
**Patient information requested:**		Most respondents indicated the need for detailed patient information from all categories. Most important is to know about ongoing care and treatment and diagnoses and illnesses of the patient.	P1:” It is important to know the goals of care. The patient may not want any life-sustaining treatment, preferring to die at home…such a plan is very important for the ambulance to know. Partly out of dignity and that all people must be able to decide for themselves. If you have decided something, but that is not fulfilled, because no one has remembered to inform about it, then the whole thing falls apart.”	Knowing the plan and goals of care can help ACs in the decision-making process, which in the bigger perspective also is related to the concept of dignity and co-determination for the patient.	**Defined goals of care** The ACs regard several different sources of information to construct an understanding of the overall situation. Comprehensive patient information, such as care plans and treatment restrictions, helps ACs ensure that care decisions reflect the patient's wishes and long-term goals. Without this information, there is a risk of misaligned care that may not meet the patient's preferences.
Ongoing care and treatment	118 (100)				
Diagnoses and illnesses	117 (99.2)				
Pharmaceutical treatment	115 (97.5)				
Treatment restrictions	114 (96.6)				
Nurse contact information	107 (90.7)				
Home care plan	105 (89)				
Summary of recent hospitalization	103 (87.3)				
Allergies	103 (87.3)				
Blood-borne infections	100 (84.7)				
**Availability of patient information: **		The availability of requested patient information is described as insufficient by the majority of ACs. Only 11 percent perceive current availability to patient information as adequate.	P12:” It's so hard to get information about the patient. I look in binders and make phone calls, but I never think I get any information, so you stand there with your papers and wonder... I don't have a journal…I don't have anything... and I'm standing there, in charge [of care] … and I have no idea”	The ACs lack access to the electronic patient record and instead search for written information and documentation in the home of the patient. If the care staff does not know the patient, the written information available in the patient’s home becomes even more important.	**Access to information** ACs often struggle with insufficient access to critical patient information, particularly during night shifts. This lack of access complicates decision-making and can lead to uncertainty in care delivery. Improved access to electronic health records and other patient data is necessary to enhance the accuracy and timeliness of ACs' decisions.
Satisfactory^a^ availability	13 (11)				
Insufficient^b^ availability	105 (89)				
**Satisfactory**^a^** collaboration with:**		Satisfactory collaboration is most often reported with the patient's family and care staff. In contrast, collaboration with the home care nurse and primary care physician is consistently rated lower.	P18:” I feel sorry for the care staff who are there when you come and want the best for the patient and are very stressed, because they don't know what to do or how to handle it. And they are often very helpful and help and load and pack and support.” P7: “Unfortunately, it is my opinion that the home care nurse is happy to leave before we arrive ... not every time .... but often…those who might be able to give us the valuable information have left”	Apart from the family, the care staff are the ones with knowledge of the everyday life of the patient. ACs describe care staff as trying to assist in any way they can, yet with limitations in knowledge or resources. The home care nurse is described as an important source of information to the ACs. Their presence on-site is frequently desired yet often unavailable.	**Clarity in roles and responsibilities** When roles are well-defined, ACs can better coordinate with other healthcare actors, leading to more streamlined and effective patient care. Conversely, unclear roles can result in confusion, delays, and compromised patient outcomes.
Patients’ spouse	108 (97.3)^c(7)^				
Patients’ children	98 (89.1)^c(8)^				
Care staff, day	102 (86.4)				
Care staff, night	94 (79.7)				
Primary care nurse	87 (75.7)^c(4)^				
Home care nurse, day	72 (61.5)^c(1)^				
Primary care physician	56 (50.5)^c(7)^				
Home care nurse, night	48 (41)^c(1)^				

^a^Satisfactory = rated 3 or higher on a scale from 1 - 4

^b^Insufficient = rated 2 or lower on a scale from 1 – 4

^c^No experience (n)

#### Defined goals of care

Defined goals of care are a significant factor influencing interprofessional collaboration. Survey data revealed that nearly all ACs considered access to a patient's care plan to be relevant. Most ACs identified ongoing care and treatment, alongside details of diagnoses, illnesses, and pharmaceutical treatments, as the most important pieces of patient information for alignment with the patient’s goals of care. Qualitative data further support these findings, underscoring the ACs’ preference for having comprehensive patient information available, to guide decision-making. Information such as treatment restrictions or desired outcomes was seen as vital for ensuring that emergency interventions aligned with the patient’s broader care plan, reflecting their preferences and long-term objectives. Conversely, the absence of documented care plans or a lack of consensus among healthcare professionals could lead to misalignment, resulting in delays and potentially compromising the quality of care provided.

#### Access to information

Access to patient information is another key factor influencing collaboration in prehospital emergency care. Survey data showed that only a small proportion of ACs felt satisfied with their current access to essential patient information, with the vast majority finding it inadequate. This widespread lack of access to critical patient information was consistently recognized as a significant challenge in delivering effective care. Qualitative insights further underscored the importance of availability and continuity of information. Continuity of information was considered vital for ensuring that all healthcare actors were on the same page regarding the patient's care needs. Many ACs reported that the availability of comprehensive patient information—such as medical records, care plans, or even basic patient details—was often limited, particularly during night shifts. This was particularly problematic in cases involving complex care needs, where a detailed understanding of the patient’s medical history and current treatment plan was essential. ACs expressed a strong desire for access to electronic health records and other sources of patient information, which would enable them to make more informed and timely decisions. The absence of such resources often forced ACs to rely on incomplete or outdated information, increasing the risk of suboptimal decision-making and the likelihood of unnecessary hospital transports.

#### Clarity in roles and responsibilities

Clarity in roles and responsibilities emerged as a key factor, with availability of key healthcare actors identified as essential for effective collaboration. When roles were unclear or essential healthcare actors were not available on site, disruptions in care and collaboration were common. Qualitative insights further highlighted the importance of knowledge and decision support systems in navigating complex care situations. Such tools helped ensure that all healthcare actors understood their responsibilities, reducing uncertainty and improving care outcomes. Conversely, when roles were not well-defined, ACs often faced confusion and delays, compromising patient care. Effective collaboration also relied on healthcare providers working together to create and implement patient care plans. This required a shared understanding of the patient’s needs and the use of both internal resources, such as team expertise, and external resources, like additional healthcare professionals or services. Building strong collaborative relationships, characterized by trust, mutual respect, and cooperation, further enhanced these efforts. Notably, collaboration in the island region was consistently rated higher than in other regions. This may be attributed to the well-developed local healthcare infrastructure and the continuity of care staff, both of which were highlighted by ACs as contributing to more effective collaboration.

## Discussion

This study aimed to explore ambulance clinicians'perspectives on interprofessional collaboration in prehospital emergency care for older patients with complex care needs and to identify key factors influencing collaboration. The findings indicate that clear communication, information availability, and well-defined roles are key factors for successful interprofessional collaboration. However, several barriers limit the effectiveness of ACs in providing care, most often related to communication and access to patient information. These barriers, along with challenges in role clarity and geographical variations in collaboration, are discussed in the context of existing literature.

One of the most significant findings of this study was the widespread dissatisfaction among ACs regarding access to patient information, with only 11% reporting their access as satisfactory. This limitation may severely hamper ACs ability to make informed decisions, particularly in cases where patients have cognitive impairments, language barriers, or are otherwise unable to communicate their medical history. ACs described feeling as though they were'working blindly'or'playing a guessing game'when essential patient details—such as medical history, medication lists, or advance care directives—were unavailable. This lack of information can lead to ethical and clinical dilemmas, especially when treatment decisions may not align with a patient’s prior preferences [85–88]. Insufficient communication—whether through written documentation, electronic health records, or verbal reports—further exacerbates these challenges, increasing the risk of inconsistent or unnecessary interventions in prehospital care. This observation aligns with previous research, which highlights how professional silos in healthcare—such as the divisions between emergency care, primary care, and municipal care—contribute to differing expectations and interpretations, increasing the risk of communication failures [89]. Effective communication is essential in avoiding misunderstandings and ensuring continuity of care, particularly in high-pressure prehospital environments [90, 91].

This study underscores the importance of effective interprofessional collaboration and information transfer to preserve the integrity and co-determination of older patients with complex care needs. The likelihood of making decisions that do not reflect the patient’s preferences increases when patient wishes are not clearly communicated through the care chain. Information and communication are vital for creating shared mental models, which improve a team’s ability to communicate and coordinate effectively [92, 93]. Structured communication tools, such as SBAR (Situation, Background, Assessment, Recommendation), could be particularly valuable in this context. SBAR is a well-established method in healthcare that supports the development of shared mental models by delivering information systematically, ensuring that it is understandable to all the parties involved [94–96]. Fully implementing such tools could bridge gaps in communication and information transfer between different healthcare actors involved in the care of older patients with complex needs.

ACs in this study expressed a high degree of dependence on other healthcare actors in the care of older patients with complex needs. The continuity of home care staff and the quality of information transfer between municipal and primary care professionals directly influenced the ACs'ability to obtain up-to-date information about a patient’s ongoing care and treatment. Deficiencies in resources within primary or municipal care settings can significantly impact the conditions under which ACs operate. This finding is particularly relevant in the context of ongoing primary care reforms in Sweden, which aim to strengthen primary care’s role in managing the needs of older patients with complex conditions. Previous research on the assessment of patients who do not require ambulance care has highlighted the need to develop and strengthen guidelines and cooperation with other healthcare providers [97]. Interprofessional collaboration among healthcare professionals with different backgrounds and perspectives is more likely to generate innovative solutions to clinical challenges [71], particularly in complex care scenarios. The challenges reported by ACs in coordinating care with home care nurses, especially during night shifts, underscore the importance of ensuring that all healthcare actors involved in patient care are readily available and have clearly defined roles.

The study also raises important questions about the evolving role of the ambulance service. The findings suggest a need for clearer role definitions within the context of a changing healthcare landscape. The role of ACs in navigating complex care scenarios is consistent with the global understanding of prehospital emergency care as a critical link in the continuum of care [19–22]. The need for clear communication of care goals and access to comprehensive patient information reflects broader challenges faced by healthcare providers in delivering coordinated care in high-pressure environments, as noted in previous studies [24–35]. This study also supports the notion that advanced clinical decision-making in prehospital settings requires not only well-defined roles but also robust decision support systems and access to comprehensive patient information. This is consistent with the broader trend towards enhancing out-of-hospital care by equipping healthcare providers with the tools and information they need to make informed decisions in real-time [49–52, 98].

The geographical analysis of the results revealed that collaboration was rated higher in more sparsely populated areas. The island region consistently reported the highest ratings, suggesting that factors unique to this region—such as well-established collaboration structures and stronger interprofessional relationships—may contribute to better teamwork and information exchange. Research has suggested that rural and remote healthcare settings often foster closer professional networks due to smaller healthcare teams and a greater reliance on collaboration for patient care [99–102]. In contrast, the north region reported the lowest ratings, indicating potential areas for improvement in interprofessional collaboration strategies. Differences in healthcare infrastructure and resource availability likely shape how interprofessional collaboration functions across geographical areas. The lack of significant differences in collaboration with family members suggests that personal interactions with patients'families are more uniformly experienced across regions. These insights can guide targeted interventions to enhance collaboration where it is needed the most. However, further studies are needed to determine whether these findings can be transfered to rural and/or urban settings.

### Clinical implications and directions for future research

The findings of this study carry significant implications for clinical practice. First, healthcare providers and policymakers should prioritize the development of systems that ensure clear communication of care goals, especially for older patients with complex care needs. This could involve the implementation of standardized care plans that are easily accessible to all relevant healthcare professionals. Second, improving access to patient information through enhanced integration of electronic health records and other information-sharing tools across healthcare settings is crucial for supporting informed decision-making in prehospital care, particularly at night. Ensuring that ambulance clinicians can access essential patient data, such as medical history, medication lists, and advanced directives, would enhance the quality and safety of care provided during emergencies. Third, clearly defining and communicating roles and responsibilities among all members of the healthcare team is essential for reducing confusion and improving collaboration. Targeted interprofessional training programs, could help address variations in collaboration satisfaction and ensure that all professionals are well-prepared to work together effectively in time-sensitive situations.

To further advance the field, future research should focus on developing and evaluating innovative information-sharing technologies, such as integrated electronic health records and mobile applications, that can improve decision-making and care coordination in prehospital settings. Comparative studies could examine different models of interprofessional collaboration across various healthcare systems, with a particular emphasis on their effectiveness in managing care for older patients with complex care needs. Longitudinal research is needed to assess the long-term effects of improved collaboration on patient outcomes and healthcare system efficiency. Additionally, investigating the roles of patients and their families in setting and communicating care goals—and how they impact collaboration and patient satisfaction—remains an important area for future inquiry. To ensure broader applicability, future studies should also be conducted across diverse regions and healthcare systems.

### Strengths and limitations

This study has several strengths, including the use of an explanatory sequential mixed-methods design, which provides a comprehensive understanding of interprofessional collaboration in prehospital emergency care. By integrating quantitative survey data with qualitative interview insights, it is possible to gain more in-depth understanding of a subject area [72, 73]. In the prehospital research field, mixed methods are a suitable choice. This is due to their potential to address healthcare issues in complex, diverse environments where both qualitative and quantitative forms of data may be relevant [103]. Although time-consuming, mixed methodology can help address broader issues and add insights that might otherwise have been missed. Notably, similar designs have proved useful in related studies on care transitions of older individuals with multimorbidity [104].

The sample of 118 participants exceeded the initial calculation of 115 participants, achieving a statistically sufficient number that represents the overall sample [105] with a 95% confidence interval. This robust response rate provided a sample across different geographical regions, ensuring a representative demographic distribution of the regional ambulance service. This enhances the reliability and validity of the study findings, confirming that the sample is representative of the broader population of ACs. The approach of purposive sampling ensured that a diverse range of ACs, representing different levels of experience and geographical areas, could contribute to the study. However, as participation was voluntary, the potential for self-selection bias cannot be ruled out. Despite the adequate sample size, the potential for response bias remains a limitation, as those who chose to participate may have different perspectives than those who did not.

In order to answer the research question from a variety of aspects [106] and promote the credibility of the sample, all occupational categories in near-patient work on ambulances were included in the initial phase of the study to investigate the outcome and the different roles'experiences. The qualitative phase, consisting of 20 detailed interviews, offered rich contextual data that deepened the understanding of the survey findings, particularly the nuances of interprofessional collaboration. Additionally, both quantitative and qualitative data underwent rigorous analysis, including descriptive statistics, chi-square tests, Fisher’s exact test, Kruskal–Wallis tests, and inductive content analysis, ensuring the robustness and reliability of the findings. The study's findings have direct implications for practice, offering actionable recommendations to improve collaboration and care for older patients with complex needs in prehospital settings.

However, the study also has limitations. The cross-sectional design captures data at a single point in time, limiting the ability to infer causal relationships or changes over time. The reliance on self-reported survey and interview data may introduce bias, as respondents might provide socially desirable answers or have varying interpretations of the questions. The use of a 4-point Likert scale [80, 81] might have restricted the depth of responses compared to 5- or 7-point scale. However, this choice was intentional to minimize neutral responses and encourage clear distinctions between perceived insufficiency and adequacy in collaboration. Additionally, some participants did not specify their geographical area, which could lead to incomplete analysis and interpretation of regional differences in collaboration experiences. The quantitative phase's sample size was relatively small, limiting the extent to which inferences could be made from the results. Although the study included a representative sample of the regional ambulance service, the findings may not be generalizable to other regions or countries with different healthcare systems and cultural contexts. While member checking could have further strengthened the credibility of the qualitative findings, it was not conducted due to the risk of placing additional burden on participants in the context of their demanding clinical roles. Instead, credibility was supported through peer debriefing, where preliminary themes and interpretations were discussed among the research team, including clinically active registered nurses, to challenge assumptions and ensure a balanced analysis.

## Conclusion

This study highlights the importance of defined goals of care, access to information, and clarity in roles and responsibilities for enhancing interprofessional collaboration in prehospital emergency care for older patients with complex care needs. The findings indicate that although ambulance clinicians value clear communication of care goals, they often face challenges due to insufficient access to essential patient information, which hinders effective decision-making. Furthermore, the variability in collaboration satisfaction, particularly with primary care physicians and home care nurses during night shifts, underscores the need for well-defined roles and improved communication pathways. To address these barriers, healthcare systems should implement structured communication protocols, enhance digital information-sharing solutions, and foster stronger interprofessional training initiatives. These measures can improve collaboration, facilitate timely decision-making, and ensure more coordinated and patient-centered care in prehospital settings.

## Supplementary Information

Supplementary Material 1.

Supplementary Material 2.

Supplementary Material 3.

## Data Availability

The datasets analyzed during the current study are available from the corresponding author on reasonable request.

## Abbreviations

AC: Ambulance clinician
EMT: Emergency medical technician
RN: Registered nurse
SBAR: Situation, Background, Assessment, Recommendation
