# Supplementary material for: Ambulance clinicians’ perspectives on interprofessional collaboration in prehospital emergency care for older patients with complex care needs: a mixed-methods study
Source: BMC Geriatr. 2025 May 30;25:394. doi: 10.1186/s12877-025-05975-w (PMC12124084; doi:10.1186/s12877-025-05975-w)
Supplement: Supplementary file 1 — Supplementary Material 1. [file 12877_2025_5975_MOESM1_ESM.pdf]

Appendix 1: Good Reporting of a mixed methods study (GRAMMS)

O'Cathain A, Murphy E, Nicholl J. The quality of mixed methods studies in health services research. J Health Serv Res Policy. 2008;13(2):92-98.

**Guideline**	**Section: page**
Describe the justification for using a mixed methods approach to the research question	Design: p.7<br>Strengths and limitations: p.34-36
Describe the design in terms of the purpose, priority and sequence of methods	Design: p.7
Describe each method in terms of sampling, data collection and analysis	Data collection: p.10<br>Data analysis: p.10-11
Describe where integration has occurred, how it has occurred and who has participated in it	Design: p.7-8
Describe any limitation of one method associated with the present of the other method	Strengths and limitations: p.34-36
Describe any insights gained from mixing or integrating methods	Discussion: p.30-33,<br>Clinical implications and directions for future research: p.34<br>Strengths and limitations: p.34-36
