# Supplementary material for: Ambulance clinicians’ perspectives on interprofessional collaboration in prehospital emergency care for older patients with complex care needs: a mixed-methods study
Source: BMC Geriatr. 2025 May 30;25:394. doi: 10.1186/s12877-025-05975-w (PMC12124084; doi:10.1186/s12877-025-05975-w)
Supplement: Supplementary file 2 — Supplementary Material 2. [file 12877_2025_5975_MOESM2_ESM.pdf]

Appendix 2: Online survey

* = Required

Your education level: *
(Dropdown list with single choice answer)

- ☐ EMT
- ☐ Registered nurse
- ☐ Registered nurse with ambulance specialization
- ☐ Registered nurse with other specialization
- ☐ Other

Experience in ambulance care: *
(Number of years)

(Free text)

Geographical area of work: *

(Free text)

What obstacles do you experience in the ambulance care for assessing and caring for older patients with complex care needs? *

(Free text)

How would you rate the collaboration with the following actors? *
(1=Insufficient, 4=Satisfactory)

	1	2	3	4	No experience
Home care staff, daytime					
Home care staff, night					
Home care nurse, daytime					
Home care nurse, night					
Primary care nurse					
Primary care physician					
Patients’ spouse					
Patients’ children					

Comment:

(Free text)

Describe your experience of interprofessional collaboration with the following actors in the care of an older person with complex care needs:

Home care staff: *

(Free text)

Municipal home care: *

(Free text)

Primary care: *

(Free text)

Would you have use for access to the patient's written care plan? *

- ☐ Yes
- ☐ No
- ☐ Maybe

Comment:

(Free text)

What information do you need to obtain in the case the patient is not able to convey this? *
(Check all that apply)

- ☐ Diagnoses and illnesses
- ☐ Pharmaceutical treatment
- ☐ Summary of recent hospitalization
- ☐ Ongoing care and treatment
- ☐ Nurse contact information
- ☐ Home care plan
- ☐ Treatment restrictions
- ☐ Allergies
- ☐ Blood-borne infections

How would you rate the access to this information today? *
(1=Insufficient, 4=Satisfactory)

1	2	3	4

Comment:

(Free text)

In your experience, what (if anything) needs to change to maintain a safe and secure care of the older person with complex care needs? *

(Free text)
