# Supplementary material for: Ambulance clinicians’ perspectives on interprofessional collaboration in prehospital emergency care for older patients with complex care needs: a mixed-methods study
Source: BMC Geriatr. 2025 May 30;25:394. doi: 10.1186/s12877-025-05975-w (PMC12124084; doi:10.1186/s12877-025-05975-w)
Supplement: Supplementary file 3 — Supplementary Material 3. [file 12877_2025_5975_MOESM3_ESM.pdf]

Appendix 3: Interview guide

Interprofessional collaboration

- What does collaboration in the care of older patients with complex care needs mean to you?
    - How do you perceive your role in this collaborative process?
- Can you describe your experiences of collaborating with primary care, municipal care, and home care staff in the care of older patients with complex care needs?
- Can you share an example of a situation where collaboration around an older patient worked well?
    - What factors contributed to making this collaboration effective?
- Can you describe a situation where collaboration was lacking?
    - What contributed to this?
    - How did you manage the situation?
- What obstacles do you encounter in interprofessional collaboration when caring for older patients with complex care needs?
    - How do you think these obstacles could be removed or managed?

Access to information/information transfer

- What types of information do you need when caring for older patients with complex care needs?
    - Why is this information important?
- How would you describe the current state of access to patient information? In what ways does this impact your work?
- What methods are currently used to transfer patient information in the prehospital setting?
- How do you obtain the patient information you need?
- What obstacles do you face in the transfer of patient information when caring for older patients with complex care needs?
    - How could these obstacles be addressed or overcome?

- Is there anything else you would like to add?
